# Use of Remotely Sensed Data to Evaluate the Relationship between Living Environment and Blood Pressure

**DOI:** 10.1289/ehp.0900871

**Published:** 2009-08-04

**Authors:** Maurice G. Estes, Mohammad Z. Al-Hamdan, William Crosson, Sue M. Estes, Dale Quattrochi, Shia Kent, Leslie Ain McClure

**Affiliations:** 1 Universities Space Research Association and; 2 Earth Science Office, NASA–Marshall Space Flight Center, Huntsville, Alabama, USA; 3 Department of Biostatistics, University of Alabama–Birmingham, Birmingham, Alabama, USA

**Keywords:** blood pressure, hypertension, living environment, remote sensing, urban

## Abstract

**Background:**

Urbanization has been correlated with hypertension (HTN) in developing countries undergoing rapid economic and environmental transitions.

**Objectives:**

We examined the relationships among living environment (urban, suburban, and rural), day/night land surface temperatures (LST), and blood pressure in selected regions from the REasons for Geographic and Racial Differences in Stroke (REGARDS) cohort. Also, the linking of data on blood pressure from REGARDS with National Aeronautics and Space Administration (NASA) science data is relevant to NASA’s strategic goals and missions, particularly as a primary focus of the agency’s Applied Sciences Program.

**Methods:**

REGARDS is a national cohort of 30,228 people from the 48 contiguous United States with self-reported and measured blood pressure levels. Four metropolitan regions (Philadelphia, PA; Atlanta, GA; Minneapolis, MN; and Chicago, IL) with varying geographic and health characteristics were selected for study. Satellite remotely sensed data were used to characterize the LST and land cover/land use (LCLU) environment for each area. We developed a method for characterizing participants as living in urban, suburban, or rural living environments, using the LCLU data. These data were compiled on a 1-km grid for each region and linked with the REGARDS data via an algorithm using geocoding information.

**Results:**

REGARDS participants in urban areas have higher systolic and diastolic blood pressure than do those in suburban or rural areas, and also a higher incidence of HTN. In univariate models, living environment is associated with HTN, but after adjustment for known HTN risk factors, the relationship was no longer present.

**Conclusion:**

Further study regarding the relationship between HTN and living environment should focus on additional environmental characteristics, such as air pollution. The living environment classification method using remotely sensed data has the potential to facilitate additional research linking environmental variables to public health concerns.

Hypertension (HTN) is a risk factor for heart disease, stroke, other cardiovascular diseases, and renal disease and has been identified as the second leading cause of diseases worldwide (Ezzati et al. 2002). It has been estimated that 26.4% of the global adult population is hypertensive, with 333 million hypertensive individuals in developed countries and 972 million hypertensive individuals in developing countries (Kearney et al. 2005). In developed countries, 10.9% of disability-adjusted life years lost has been attributed to HTN.

Cardiovascular health issues relating to urbanization are of particular concern because in this century the world has experienced an unprecedented urban growth, with > 50% of the world’s population residing in cities and mega-cities (Godfrey and Julien 2005). Urbanization has been correlated with HTN in developing countries undergoing rapid economic and environmental transitions, such as China, India, and many African countries (Kusuma and Das 2008; Opie and Seedat 2005). The United States, a large, ethnically diverse, and relatively wealthy country, has a vastly different distribution of HTN risk factors compared with both developing countries and most other developed countries, but urban–rural differences remain an under-researched issue (Gillum 1996; Gillum et al. 2004; Obisesan et al. 2000). In the United States and worldwide, there remains the question of how much the urban environment contributes as an independent risk factor for blood pressure differences, and how much is attributable to a variety of environmental, lifestyle, and demographic correlates of urbanization (Ala et al. 2004; Nirmala 2001; Sobngwi et al. 2004). In particular, race and ethnicity are often involved in urban–rural blood pressure differences (Appel et al. 2002; Ruixing et al. 2006).

Many studies have used remotely sensed data for land cover/land use (LCLU) classification of urban areas (Jacquin et al. 2006; Kampouraki et al. 2006; Lu and Weng 2005; Lu et al. 2008; Xu and Gong 2007). However, very few studies in the United States have evaluated how these LCLU classifications and living environments affect human health. In this study we explore the relationship between urban, suburban, and rural land classifications and selected correlates with blood pressure among participants of the large, well-characterized African-American and white cohort from the REasons for Geographic and Racial Differences in Stroke (REGARDS) study. This innovative study used remote sensing data to apply LCLU techniques to classify the geo-coded REGARDS participants. LCLU urban classification has been used extensively in environmental studies; however, its application to public health research is rare and represents an innovative opportunity to explore this and potentially many other public health issues.

We examined the relationship between living environment (defined as rural, suburban, and urban) and day/night (maximum and minimum) land surface temperature (LST), and blood pressure in persons from the REGARDS cohort living in selected regions of the United States. We hypothesized that elevated blood pressure may be a function of living environment. In the past, urbanization classification was based on U.S. Department of Agriculture rural–urban continuum codes. Our study used remote sensing spatial data to classify rural, suburban, and urban areas to examine differences in living environment and blood pressure measurements. Our first objective was to examine the relationship between rural, suburban, and urban residents and blood pressure [measured as systolic blood pressure (SBP), diastolic blood pressure (DBP), and HTN] to determine whether higher blood pressure is associated with living environment after adjusting for known risk factors. The second objective was to examine differences in LST for each category of living environment for the 2 weeks before the blood pressure measurement to validate expected temperature variations between the living environment classes and to determine if higher LST is associated with higher blood pressure.

## Materials and Methods

**Figure 1:**
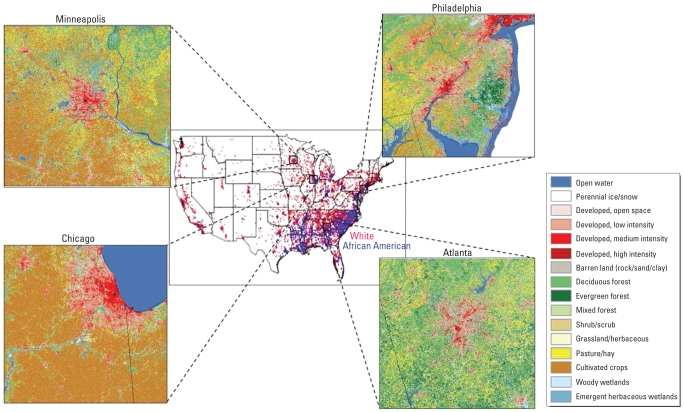
REGARDS study national distribution and 30-m NLCD-2001 LCLU information for the four study areas: Chicago, Minneapolis, Philadelphia, and Atlanta. Black dot marks the center of the city downtown.

Four geographically and climatologically distinct U.S. regions that include major urban centers and that have varying stroke mortality rates were selected for this study. These regions are a southeastern region centered on Atlanta, Georgia; a northeastern region centered on Philadelphia, Pennsylvania; and two Midwestern regions centered on Chicago, Illinois, and Minneapolis, Minnesota. Stroke mortality is lowest among residents of Minnesota, followed by residents of Pennsylvania, then residents of Illinois, and highest among residents of Georgia (Casper et al. 2003). General land use configurations are similar; however, Philadelphia and Chicago have higher density and more compact urban areas, whereas Atlanta and Minneapolis have more fragmented urban development patterns. Significant differences in vegetation and local climatology also exist among the regions. The regions are approximately 200 km × 200 km, which allowed for significant populations in rural, suburban, and urban locations to be evaluated, as shown in Figure 1.

The study is an urban to subregional scale investigation that uses 1-km data products from NASA (National Aeronautics and Space Administration) Moderate-Resolution Imaging Spectroradiometer (MODIS) for day and night LST and 30-m LCLU information from the 2001 National Land Cover Data (NLCD-2001). These data have been compiled on a 1-km grid for the four regions selected for analysis. The environmental data have been matched with the REGARDS data via an algorithm that uses the spatial location of the participants’ residences.

### Description of REGARDS

The REGARDS study is a national population-based cohort study that recruited 30,228 participants ≥ 45 years of age, with 45% male, 55% female (goal was 50% male, 50% female), and 42% African American, 58% white (goal was 50% African American, 50% white). The national distribution of the REGARDS cohort is shown in Figure 1. Recruitment of the cohort began in January 2003 and was completed in October 2007. Twenty-one percent of the cohort was recruited from the “buckle” of the stroke belt (coastal plain region of North Carolina, South Carolina, and Georgia), 35% from the stroke belt states (remainder of North Carolina, South Carolina, and Georgia, plus Alabama, Mississippi, Tennessee, Arkansas, and Louisiana), and the remaining 44% from the other 42 contiguous states (goal was 20% buckle, 30% belt, 50% remainder of nation). The methods have been published elsewhere (Howard et al. 2005). Participants were selected from commercially available lists of residents, using a combination of mail notification and telephone contact. Baseline data, including demographics, cardiovascular risk factor history, and others, were collected via computer-assisted telephone interview, at which time verbal consent was obtained. After completion of the baseline interview, an in-home visit was performed to collect physical measurements, including blood pressure, a blood sample, and a urine sample; at this time signed informed consent was obtained. Follow-up phone contact is made at 6-month intervals to assess occurrence of stroke and cardiac events. The study was approved and monitored by the institutional review boards at all participating institutions.

Blood pressure was determined during the in-home visit as the average of two seated measurements. HTN was defined as SBP > 140 mmHg, DBP > 90 mmHg, or self-reported use of antihypertensive medications. Age, sex, race, income, and education were determined by self-report, whereas weight and height were evaluated during the in-home visit. REGARDS participants were geocoded using SAS/GIS (geographic information system) geocoding software (version 9.1; SAS Institute Inc., Cary, NC; SAS Institute Inc. 2000), based on the street address of the participant and were then matched by census tract to community-level poverty, expressed as the proportion of the census tract below the poverty level (0–5%, 5–10%, 10–25%, or > 25%). SAS/GIS generates a score describing the likelihood that the provided longitude and latitude are matched identically to the address, and 88% of the participants included in these analyses had a score of ≥ 80%.

### Landsat and MODIS remotely sensed data

The Landsat satellite program has continuously gathered information on changes in Earth’s landscape since the 1970s (NASA 2009). Since 1999, the Landsat 7 satellite has been collecting visible and infrared data on Earth’s surface at spatial resolutions from 15 to 60 m. These data were used to develop the NLCD-2001 used in this project as a baseline from which to determine living environments. The NLCD-2001 product at 30-m spatial resolution represents land cover based on Landsat 7 data from 1999–2003 and provides 16 LCLU classes (an additional four classes are available in Alaska only and another nine classes in coastal areas). All NLCD-2001 products were generated according to protocols outlined in Homer et al. (2004) using 66 mapping zones for the conterminous United States and 23 zones in Alaska. Formal accuracy assessment of the NLCD-2001 products is planned in the near future based on the design outlined in Stehman et al. (2008). However, users can gain initial feedback on product accuracy from the cross-validation estimate of product accuracy provided from the algorithms employed in NLCD-2001 modeling (Homer et al. 2007). Cross-validation accuracy of the land cover product was weighted by class occurrence in each mapping zone. Accuracy estimates across mapping zones ranged from 70% to 98%, with an overall average accuracy across all zones of 83.9% (Homer et al. 2007).

The MODIS instrument is carried by the Aqua satellite, which views most of the earth’s surface twice daily, at nominally 0130 hours and 1330 hours local time. The instrument provides a validated LST product in Kelvin on a 1-km grid for a pair of daytime and nighttime observations. Global observations are available from 2000 to the present. This product is the result of the generalized split-window LST algorithm developed by Wan and Dozier (1996). Comparisons between data from this LST product and *in situ* values in 47 clear-sky cases, which were made in a study by Wan (2008), indicated that the accuracy of this product is better than 1 K in 39 of 47 cases. The root mean squared difference was determined to be < 0.7 K.

### Method for urban, suburban, and rural delineations

A methodology was developed using the NLCD-2001 LCLU grids over the study area to delineate rural, suburban, and urban zones and resample to a 1-km grid. The NLCD-2001 classification contains four developed classes: developed high intensity, developed medium intensity, developed low intensity, and developed open space. The developed high-intensity class is consistent with urban living near the central business districts and other highly urbanized land use areas containing a mixture of commercial, industrial, and residential land uses. Condominiums, apartment complexes, and row houses are typical living environments in the developed high-intensity class. Conversely, the developed open-space class commonly includes very low-density suburban environments with large-lot single-family housing units along with parks and golf courses. The developed low-intensity and medium-intensity classes are similar in that both contain single-family housing units in conjunction with low to moderate levels of urban development, with the main difference being the average size of the housing lots.

**Figure 2:**
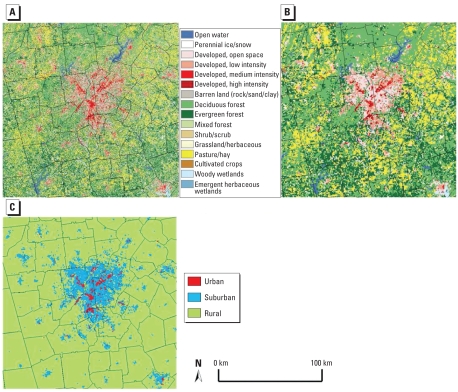
Demonstration of the resampling and urban, suburban, and rural delineation methodology (Atlanta): 30-m NLCD-2001 (*A*), 1-km resampled NLCD-2001 using most dominant classification (*B*), and living environment category (urban, suburban, rural) at 1 km (*C*).

Based on the land use characteristics of the developed classes, a remapping scheme was developed to map the developed high-intensity and developed medium-intensity classes to the urban living environment class and the developed low-intensity and developed open-space classes to the suburban living environment class. All other NLCD-2001 classes, such as forest and agriculture classes, are included in the rural living environment, as demonstrated in Figure 2.

### Dominant class algorithm role in methodology

Resampling is the process of assigning, interpolating, or extrapolating new cell values when transforming raster data, or images, to a new coordinate space or cell size (i.e., spatial resolution). To be consistent with the MODIS LST data spatial resolution and because people spend most of their time outside a 30 m × 30 m area, we resampled the raw NLCD-2001 data in this study from 30 m to 1 km in order to evaluate the LCLU and living environment effects on SBP, DBP, and HTN. We also analyzed data at a 3-km scale to study the effect of scale on such potential relationships. We developed a resampling algorithm that uses the raw data set (30-m NLCD-2001), calculates the areas of all the LCLU classes within each coarse 1-km or 3-km grid cell (filter window), and assigns the dominant LCLU class to that coarse grid cell in a GIS, as demonstrated in Figure 2 for the city of Atlanta.

### LST data processing

To determine temperature effects on SBP, DBP, and HTN, LST data from the MODIS sensor aboard NASA’s Aqua satellite were obtained for the four focus cities for the period 2003–2007. Both day (~ 1330 hours) and night (~ 0130 hours) data were obtained. The MODIS LST product is produced only for land surfaces determined by an algorithm to be cloud-free. Therefore, data are missing for many days and locations.

### Data linkage method

**Figure 3:**
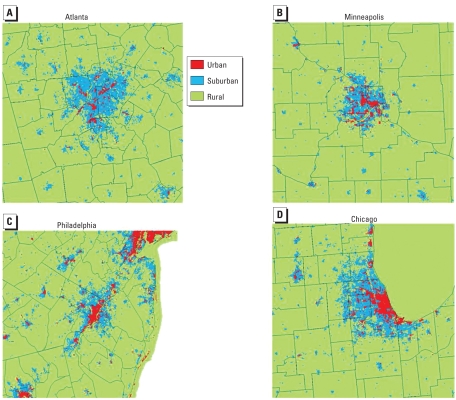
Living environment categories at 1 km for Atlanta (*A*), Minneapolis (*B*), Philadelphia (*C*), and Chicago (*D*).

After delineating the living environments as rural, suburban, or urban for the four 200 km × 200 km study areas as shown in Figure 3, and in order to assess the living environment effects on SBP, DBP, and HTN, the REGARDS participants located within those study areas were spatially linked to the living environment categories in a GIS. Given the geographic coordinates of the REGARDS participants’ residences, those participants were assigned to the living environment category of the grid cell within which they reside. That linkage process was done at three different spatial resolutions (30 m, 1 km, and 3 km) to study the effect of scale on that assessment.

Day and night LST data were processed separately in conjunction with geographic data on the residence locations of the REGARDS participants in the four cities. LST data were extracted for the grid cell for which the centroid was closest to the residence location of each subject. This was done for each day and night of the 5-year study period, creating tables of LST linked to the list of REGARDS participants. These tables provide the daytime and nighttime LST observations, when available, for each day and each subject, facilitating temporal analysis of these data together with the blood pressure data.

### Statistical analysis

Descriptive statistics are expressed overall and by living environment (rural, suburban, or urban). We used analysis of variance or chi-square tests of association to determine differences in baseline characteristics of the population by living environment classification. We used linear regression to determine whether an association exists between each of SBP and DBP, and living environment. Initially, a univariate model was run, followed by a model adjusted only for race, then a model adjusted for race (African American or white), sex (male or female), age, body mass index (BMI), income (self-reported household income), education (less than high school, high school degree, some college, college degree or greater), city of residence, and community-level poverty (based on census tract). The risk factors were selected based on previously published reports linking them to HTN. City of residence was included in the model to account for characteristics particular to the city, which may confound the relationship between living environment and SBP and DBP. The relationship between the presence of HTN and living environment classification was modeled using logistic regression, and modeling progressed in the same fashion as for both SBP and DBP (univariate, age adjusted, and multivariable adjusted). An additional model was run assessing the effect of including average LST over the 2 weeks before the in-home visit on the multivariable model. We used 2-week averages of available data to allow more observations to be included in the analysis, because LST was frequently missing (as described above).

Data were analyzed using a 1-km scale for the living environment classes, but all analyses were repeated using classifications based on both the 30-m scale and the 3-km scale. Further, comparisons were made between the proportions of subjects residing in a specific living environment class, depending on the scale used to determine the classification, to assess how the scale would influence the living environment.

## Results

### Classification and resampling of living environments

**Figure 4:**
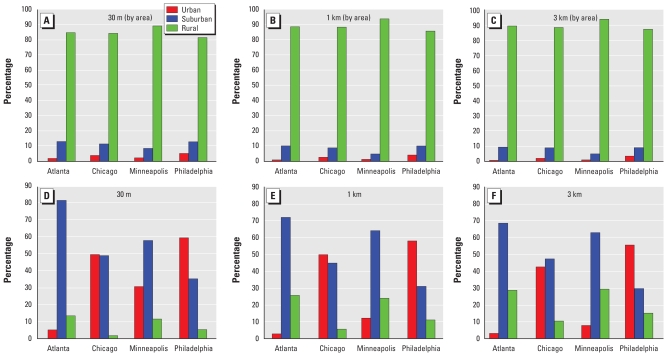
Percentage of areal coverage (*A–C*) and participants (*D–F*) of each living environment for Atlanta, Minneapolis, Philadelphia, and Chicago at three spatial resolutions.

To evaluate the effect of the scale or spatial resolution on characterizing the living environments of the REGARDS participants and later on their potential relationship with SBP, DBP, and HTN, we first calculated the percentage of areal coverage of each living environment for the four study areas and at the three spatial resolutions. As shown in Figure 4A–C, the dominant living environment over the spatial coverage of each study area was rural, with percentages > 80% at all spatial resolutions including the 30-m raw resolution. The Minneapolis study area has the highest rural and lowest urban and suburban spatial coverage. The rural living environment domination increases in all study areas as the spatial resolution decreases from 30 m down to 1 km and 3 km and the LCLU map becomes smoother.

We also determined the distribution of the REGARDS participants within each of the four study areas for the three spatial scales. As shown in Figure 4D–F, the living environments for most (> 55%) of the REGARDS participants in Atlanta and Minneapolis were characterized as suburban at all scales. On the other hand, the living environments for most of the REGARDS participants in Chicago and Philadelphia were characterized as urban. We calculated Moran’s *I* (spatial autocorrelation statistic) for the 1-km living environment research data set. In Atlanta and Minneapolis the results reflect the spatial designs or structures of those cities that tend to be more scattered (Moran’s *I* = 0.642 and 0.636, respectively), and in Chicago and Philadelphia, those that tend to be more clustered (Moran’s *I* = 0.706 and 0.869, respectively). In all cases when moving from urban or suburban classes, the percentage of participants in rural living environments increased as the spatial resolution decreased. Minneapolis was the only case where when moving from urban areas, the percentage of participants in suburban living environments increased as the spatial resolution decreased, due to the increased scatter of the urban classes compared with the other study areas.

Table 1 shows how the participants would be reclassified moving from one scale to another. Between 1 km and 3 km, there is little change in the classification of the participants: 81% of the participants remain in the same classification. The most divergent changes observed were from rural to urban (0.15%) and urban to rural (1.5%), neither of which occurred frequently. The other changes, from either rural or urban to suburban, or from suburban to either rural or urban, are more plausible and account for the remaining 18% of the changes.

In examining the change in classification when moving from a 1-km scale to a 30-m scale, there is more variation in the reclassification of the participants. Only 63% of the participants remain in the same classification at 30 m as they were for 1 km. Twelve percent change from rural to suburban, whereas 10% change from suburban to urban, and 8% change from urban to suburban. The more divergent changes, from rural to urban (2%) and from urban to rural (0.43%), happen more frequently than between the 1-km and 3-km scale, but still not often.

### LST data analysis

**Figure 5:**
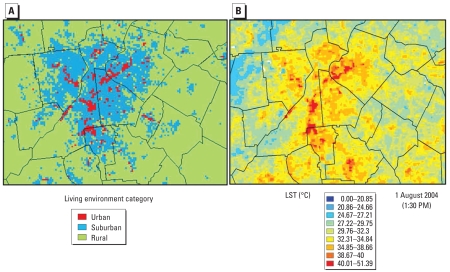
Atlanta living environment category at 1 km (*A*) versus MODIS daytime LST on 1 August 2009 (*B*).

We averaged daytime and nighttime LST data for the grids covering each of the four cities by month for each of the three living environments: rural, suburban, and urban. This analysis, performed at the 1-km scale, demonstrated that grid cells classified as urban were warmest, and rural grid cells were the coolest, as shown in Figure 5 for 1 August 2004 in Atlanta as an example. The largest difference was between suburban and rural living environments, where the mean difference, averaged over the entire 5-year period and for all cities, was 2.2°C during the day (~ 1330 hours) and 1.3°C at night (0130 hours). Results were similar among cities; the largest urban– suburban difference was in Philadelphia, and the largest suburban–rural difference was in Chicago. These results are consistent with work previously performed using aircraft remotely sensed data collected in May 1997 for the Atlanta metropolitan area that showed warmer temperatures in the central business district compared with midtown residential areas (Quattrochi et al. 2000). By revealing an “urban heat island effect” as illustrated in Figure 5, these results provide a first-order validation of the land use–based living environment classification.

### Linked data analysis

Table 2 presents the distribution of the baseline characteristics of the population, both overall and by living environment classification, based on the 1-km scale. Most of the population resides in suburban areas (52%), with almost a third in urban areas (32%), and the remaining 16% in rural areas. Those in urban areas had higher SBP and DBP than did those in suburban or rural areas, and also a higher incidence of HTN. Urban dwellers were also slightly older, more likely to be female, and more likely to be African American. The proportion of urban residents with a college degree or higher was lower than for rural or suburban areas. Residents of urban areas were more likely to have lower income, and a higher proportion of an urban census tract lived below the poverty line.

Table 3 presents the results of the univariate and multivariable modeling for SBP, DBP, and presence/absence of HTN. In the univariate model, those in urban areas had higher SBP and DBP and were more likely to be hypertensive compared with those in suburban or rural areas (all *p* < 0.0001). However, after adjustment for race, only SBP was significantly higher among those in urban than in rural or suburban areas (*p* = 0.0021), and after multivariable adjustment, even SBP was no longer significantly different across living environment classes. Adding average LST from the 2 weeks before the in-home visit to the model did not influence the results (data not shown). These results remain consistent, regardless of the scale used for the classification algorithm.

## Discussion

We found that among occupants of the four selected cities in the REGARDS study, those residing in urban areas had the highest blood pressure (both systolic and diastolic) and were more likely to have HTN compared with their counterparts in rural or suburban dwellings. However, adjustment for race and further adjustment for other known cardiovascular risk factors attenuated this association, such that these factors were no longer significantly different. This indicates that it is likely that observed differences in blood pressure by living environment classification are attributable to the distribution of race and other cardiovascular risk factors across the classes.

The development of a methodology to delineate LCLU classes into rural, suburban, and urban regions should benefit future research relating to the impact of urbanization on public health. Landsat and MODIS LCLU data are available for all areas of the United States and most areas of the world. Standard GIS software and tools used herein should be readily replicable for use in other applications. This methodology, in conjunction with remote sensing data, offers the potential to characterize physical environment features for comparison with public health data to determine correlations in multiple areas of interest.

The interpretation of results should take into account several study limitations. Although the four metropolitan study areas are diverse, the limited geographic scope did not include any Rocky Mountain, desert, or West Coast areas, so extrapolation of findings to these regions will be more difficult to substantiate. The study also considered only one public health concern, blood pressure, whereas living environment may contribute to a variety of other public health issues. Finally, the REGARDS data included only one visit during which blood pressure was measured, making temporal analysis of living environment influences unfeasible.

### Future work

The REGARDS database offers a unique and valuable opportunity to perform additional research to investigate correlations between environmental conditions, HTN, and strokes. With additional temporal data points, further evaluation of blood pressure levels and/or stroke events and correlations with either living environment or other environmental variables such as temperature or humidity could be evaluated. Further studies in geographic areas unique to this study are desirable and would make the results more robust and potentially useful for environmental public health tracking and possibly for establishing public policy (National Research Council 2007).

The National Research Council’s Earth Science and Applications from Space: National Imperatives for the Next Decade and Beyond (National Research Council 2007) encourages continued research to firmly establish the predictive relationships between remotely sensed environmental data and patterns of environmentally related health effects. Additional exploration of the uses of remotely sensed data to provide environmental data for linkage to various types of public health data is needed to gain more understanding of the potential for remotely sensed data to benefit public health research.

## Figures and Tables

**Table 1:** Changes in living environment classification resulting from spatial scale changes from 1 km to 3 km, and from 1 km to 30 m [no. (%)].

Variable	1 km → 3 km	1 km → 30 m
No change	2,665 (81)	2,071 (63)
Rural to suburban	117 (3.5)	380 (12)
Rural to urban	6 (0.15)	67 (2)
Suburban to rural	198 (6)	159 (5)
Suburban to urban	99 (3)	310 (10)
Urban to suburban	138 (4)	274 (8)
Urban to rural	47 (1.5)	14 (0.43)

**Table 2:** Distribution of SBP, DBP, and demographic factors, overall and by land use classification (scale = 1 km).

Variable	Overall (*n* = 3,298)	Urban (*n* = 1,058, 32%)	Suburban (*n* = 1,715, 52%)	Rural (*n* = 525, 16%)	*p*-Value
SBP [mmHg (mean ± SD)]	128 ± 17	131 ± 19	127 ± 17	127 ± 18	< 0.0001
DBP [mmHg (mean ± SD)]	77 ± 10	78 ± 10	77 ± 10	76 ± 10	< 0.0001
Hypertensive [no. (%)]	1,996 (61)	700 (67)	1,016 (60)	280 (54)	< 0.0001
Age [years (mean ± SD)]	65.3 ± 9.2	66.2 ± 9.2	65.0 ± 9.3	64.5 ± 8.8	0.0002
Male [no. (%)]	1,502 (46)	434 (41)	811 (47)	257 (49)	0.0012
African American [no. (%)]	1,878 (57)	871 (82)	860 (50)	147 (28)	< 0.0001
City [no. (%)]					< 0.0001
Atlanta	1,298 (39)	34 (3)	934 (55)	330 (63)	
Philadelphia	1,050 (32)	609 (58)	326 (19)	115 (22)	
Minneapolis	156 (5)	19 (2)	100 (6)	37 (7)	
Chicago	794 (24)	396 (37)	355 (21)	42 (8)	
Education [no. (%)]					< 0.0001
< High school	428 (13)	206 (20)	178 (10)	44 (8)	
High school degree	791 (24)	266 (25)	406 (24)	119 (23)	
Some college	910 (28)	299 (28)	456 (27)	155 (30)	
> College	1,164 (35)	285 (27)	673 (39)	206 (39)	
Income [no. (%)]^a^					< 0.0001
< $20,000	576 (20)	287 (30)	228 (15)	61 (13)	
$20,000–35,000	773 (26)	291 (31)	366 (24)	116 (25)	
$35,000–75,000	1,015 (35)	274 (29)	580 (38)	161 (34)	
> $75,000	570 (19)	98 (10)	340 (22)	132 (28)	
Community poverty [no. (%)]^b^					< 0.0001
0–5%	615 (21)	381 (43)	218 (14)	16 (4)	
5–10%	1,017 (35)	359 (40)	532 (34)	126 (29)	
10–25%	671 (23)	102 (11)	435 (28)	134 (31)	
> 25%	590 (20)	50 (6)	384 (25)	156 (36)	
BMI (mean ± SD)	29.2 ± 6.2	29.6 ± 6.4	29.1 ± 6.1	28.8 ± 5.8	0.036
BMI category [no. (%)]					0.24
≤ 25	803 (25)	247 (24)	418 (25)	138 (27)	
25–30	1,190 (37)	370 (35)	630 (38)	190 (37)	
> 30	1,233 (38%)	427 (41)	623 (51)	183 (36)	
Census classification [no. (%)]					< 0.0001
Rural (≤ 25% urban)	359 (11)	120 (11)	99 (6)	140 (27)	
Mixed (25–75% urban)	153 (5)	1 (< 1)	53 (3)	99 (19)	
Urban (≥ 75% urban)	2,785 (85)	937 (89)	1,562 (91)	286 (55)	

a Missing 364.

b Missing 405.

**Table 3:** Relationship between land use classification and SBP, DBP, and HTN (mmHg; scale = 1 km).

Living environment	Model 0^a^	Model 1^b^	Model 2^c^
Mean SBP			
Urban	131 ± 0.54	130 ± 0.58	128 ± 0.81
Suburban	127 ± 0.42	127 ± 0.42	127 ± 0.61
Rural	127 ± 0.76	128 ± 0.77	127 ± 0.99
*p*-Value	< 0.0001	0.0021	0.20
Mean DBP			
Urban	78 ± 0.31	77 ± 0.33	77 ± 0.47
Suburban	77 ± 0.24	77 ± 0.24	77 ± 0.35
Rural	76 ± 0.44	76 ± 0.45	76 ± 0.57
*p*-Value	< 0.0001	0.28	0.71
HTN			
Urban	1.7 (1.4–2.1)	1.2 (0.92–1.5)	1.2 (0.85–1.6)
Suburban	1.3 (1.1–1.6)	1.1 (0.89–1.3)	1.1 (0.84–1.4)
Rural	Reference	Reference	Reference
*p*-Value	< 0.0001	0.47	0.62

Values are mmHg ± SE or OR (95% confidence interval).

a Univariate.

b Adjusted for race.

c Adjusted for race, sex, age, BMI, income, education, city of residence, community level poverty.
